# Doped Non-Ti MXenes for Supercapacitor Applications

**DOI:** 10.3390/nano16191224

**Published:** 2026-09-28

**Authors:** Ramaswamy Sandeep Perala, Radha Gupta, Chandan Kumar Maity

**Affiliations:** 1 Department of Chemistry, Gachon University, 1342 Seongnam-daero, Sujeong-gu, Seongnam-si 13120, Republic of Korea; 2 Center for Multidimensional Carbon Materials (CMCM), Institute for Basic Science (IBS), Ulsan 44919, Republic of Korea

**Keywords:** heteroatom doping, doped non-Ti MXenes, electronic structure, defect chemistry, supercapacitors, structure–performance relationships

## Abstract

The rapid development of high-performing energy storage technologies has increased the search for 2D electrode materials with tunable electronic structures, abundant electroactive sites, and rapid ion transportation. Although Ti-based MXenes have been extensively investigated, non-Ti MXenes offer a broader compositional space and distinctive physicochemical properties, yet their supercapacitor applications remain comparatively underexplored. In this review, we critically examine the emerging field of doped non-Ti MXenes for advanced supercapacitor applications, including Mo-, V-, Nb-, Cr-, W-, Zr-, Hf-, Ta-, Sc-, and Y-based systems. In this review, we establish the structural, chemical, and electrochemical characteristics of non-Ti MXenes and subsequently discuss M-, X-, and T-site doping, substitution, and multielement engineering strategies. We have discussed how heteroatom doping regulates electronic structure, charge distribution, defect chemistry, interlayer spacing, surface chemistry, and ion–accessible active sites. These modifications are correlated with their influence on electrochemical kinetics, capacitance, rate capability, and cyclic stability. Finally, computational and data-driven approaches, critical benchmarking, and critical challenges are integrated to establish structure–doping–mechanism–performance relationships and design principles for next-generation doped non-Ti MXene electrodes for supercapacitors.

## 1. Introduction

The rapid growth of portable electronics, electric transportation, and renewable-energy technologies has increased the demand for energy-storage systems with high power, fast charging, and long service life. Supercapacitors are attractive for these applications because they can deliver high power density, rapid charge–discharge, and excellent cyclic stability [1,2]. Their relatively low energy density, however, remains a major limitation. The development of electrode materials with high electrical conductivity, accessible active sites, short ion–transportation pathways, and reversible redox activity is therefore important for improving their overall performance [3,4].

Two-dimensional (2D) materials have received considerable attention as supercapacitor electrodes because their thin structures provide large exposed surfaces and short pathways for electron and ion transport [5,6,7]. MXenes have emerged as an especially important class of 2D materials since the discovery of Ti_3_C_2_ in 2011 [8]. MXenes are transition–metal carbides, nitrides, and carbonitrides generally represented by M*_n_*_+1_X*_n_*T_x_, where M is an early transition metal (groups 3 to 6 elements), X is C and/or N, T_x_ represents surface terminations such as –O, –OH, –F, and –Cl, and *n* = 1–4 [9]. They are commonly obtained by selective etching of the A layer from MAX-phase precursors followed by delamination [10]. The combination of high electrical conductivity, hydrophilic surfaces, tunable surface chemistry, and layered structures makes MXenes promising for supercapacitors [11]. Their charge storage can involve both electric double-layer and pseudocapacitive processes, depending on the MXene composition, surface terminations, electrolyte, and electrode structure [12]. However, pristine MXenes suffer from several limitations, including nanosheet restacking, restricted ion accessibility, surface oxidation, limited electroactive sites, and insufficient control over their electronic structures [13]. These limitations have motivated strategies such as interlayer engineering, defect creation, surface functionalization, heterostructure formation, and elemental doping [14].

Most MXene studies have focused on Ti-based materials, particularly Ti_3_C_2_T_x_ [15,16]. Although these materials have provided important advances, the strong emphasis on Ti has left much of the broader MXene compositional space unexplored. Non-Ti MXenes based on Mo, V, Nb, Cr, W, Zr, Hf, Ta, Sc, and Y provide different electronic structures, bonding environments, redox properties, and surface chemistries [17]. The experimentally investigated systems are dominated by V-, Mo-, and Nb-based MXenes, whereas Cr-, Ta-, Hf-, Sc-, Y-, W-, and Zr-based systems remain less developed and are often supported mainly by theoretical studies [17]. The broader chemical diversity of these materials provides an opportunity to design MXene electrodes beyond the conventional Ti-based platform. The review summarizes the progression of doped non-Ti MXenes, highlighting the development of diverse compositions, M-, X-, and T-site engineering strategies, doping-induced structure–property modulation, electrochemical improvements, and emerging applications. The properties of non-Ti MXenes can be further modified through doping, substitution, and surface engineering [17,18,19]. Doping can alter the local atomic environment and influence electronic structure, charge distribution, lattice distortion, defects, surface chemistry, and interlayer spacing. The available strategies can broadly be divided into lattice substitution, functional substitution, and surface adsorption. Doping can also be introduced through in-situ and ex-situ approaches. In-situ strategies introduce the dopant during MAX-phase formation, whereas ex-situ methods modify the MXene after its synthesis [17,20,21]. The choice of approach determines the location and distribution of the dopant and therefore has a strong influence on the resulting properties.

A key issue is the distinction between doping and substitution. The terms are often used interchangeably even though they can describe different compositional regimes and structural effects. The experimental M-site doping remains difficult, while X- and T-site modifications are considerably less explored [22]. These limitations are particularly relevant because most theoretical studies have focused on metal substitution at the M site, whereas experimental verification remains limited [17]. Thus, establishing reliable synthesis methods and accurately determining dopant concentration, location, bonding, and distribution are essential steps toward understanding doped non-Ti MXenes. Doping is particularly attractive for supercapacitor applications because small changes in composition can strongly influence charge-storage behavior. Depending on their incorporation site and chemical state, heteroatom modification can alter surface termination, electronic structure, defect chemistry, ion accessibility and in case of lattice incorporation, the local structure and interlayer environment [23]. These changes can improve electrolyte accessibility, ion diffusion, charge-transfer kinetics, and pseudocapacitive reactions. Therefore, the performance of a doped MXene cannot be understood only from its chemical composition; it must be related to the interaction among dopant, host structure, surface termination, electrolyte, and charge-storage mechanism. DFT calculations can identify favorable dopant sites, formation energies, electronic structures, and ion–adsorption behavior, but many theoretically predicted doped non-Ti MXenes have not yet been experimentally realized [17,24]. Experimental studies also need better control over dopant concentration and spatial distribution.

**Figure 1:**
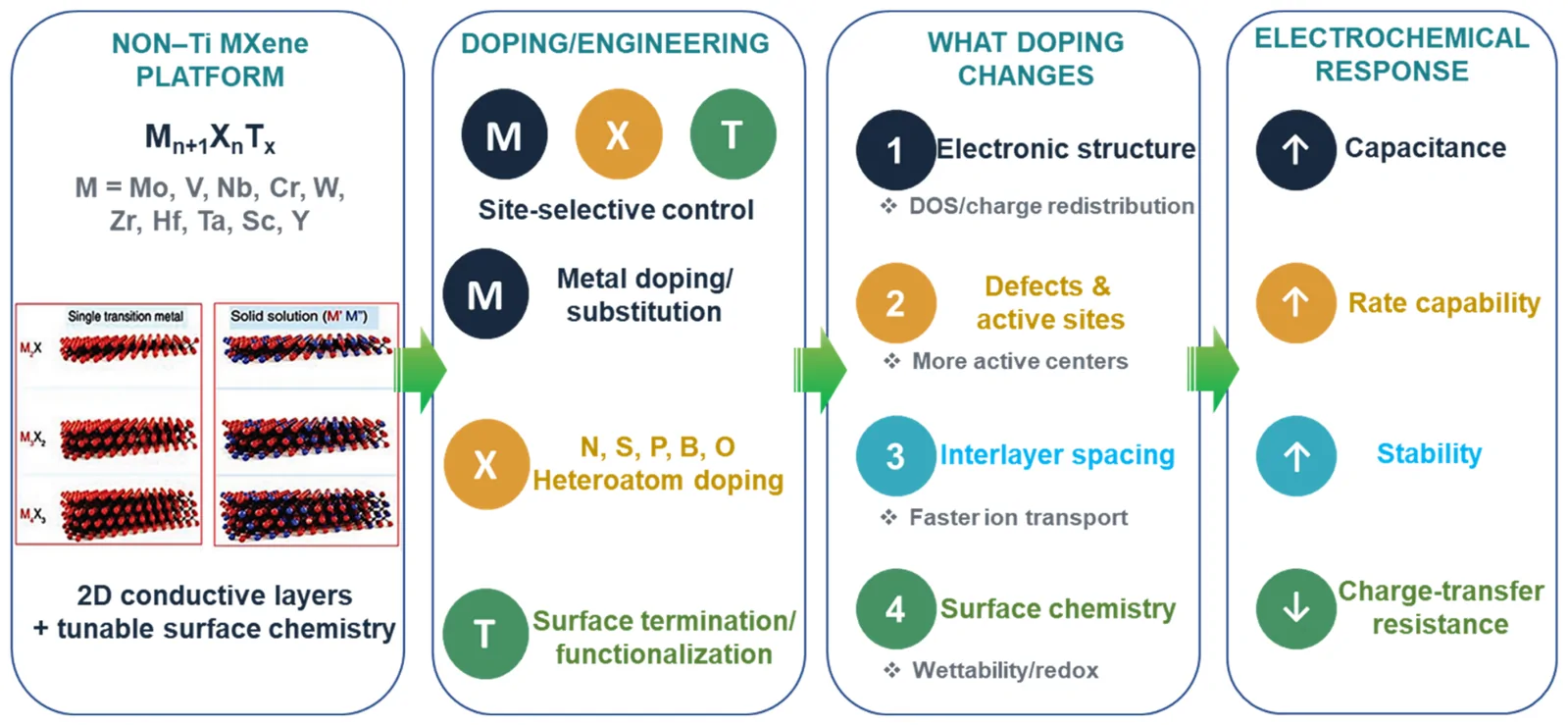
Schematic illustration of the overview for this review article.

Despite rapid progress, a focused analysis of doped non-Ti MXenes specifically for supercapacitors is still needed. Existing reviews have mainly addressed non-Ti MXenes across broad applications or doped MXenes without restricting the discussion to non-Ti systems. The present review therefore follows a structure–property–mechanism approach. First, the structural, chemical, electronic, and electrochemical characteristics of non-Ti MXenes are introduced. Next, M-, X-, and T-site doping, together with in situ, ex situ, substitutional, and surface-engineering strategies, are discussed. The effects of doping on electronic structure, defects, interlayer spacing, surface chemistry, and ion–accessible active sites are then correlated with electrochemical kinetics, capacitance, rate capability, energy/power density, and cyclic stability for supercapacitor applications. Finally, unresolved challenges and future design principles are considered. This framework aims to establish clearer structure–doping–mechanism–performance relationships and provide practical guidance for designing next-generation doped non-Ti MXene electrodes for high-performance supercapacitors. The overview of this review article is schematically shown in Figure 1.

## 2. Fundamentals of Different Non-Ti MXenes

**Figure 2:**
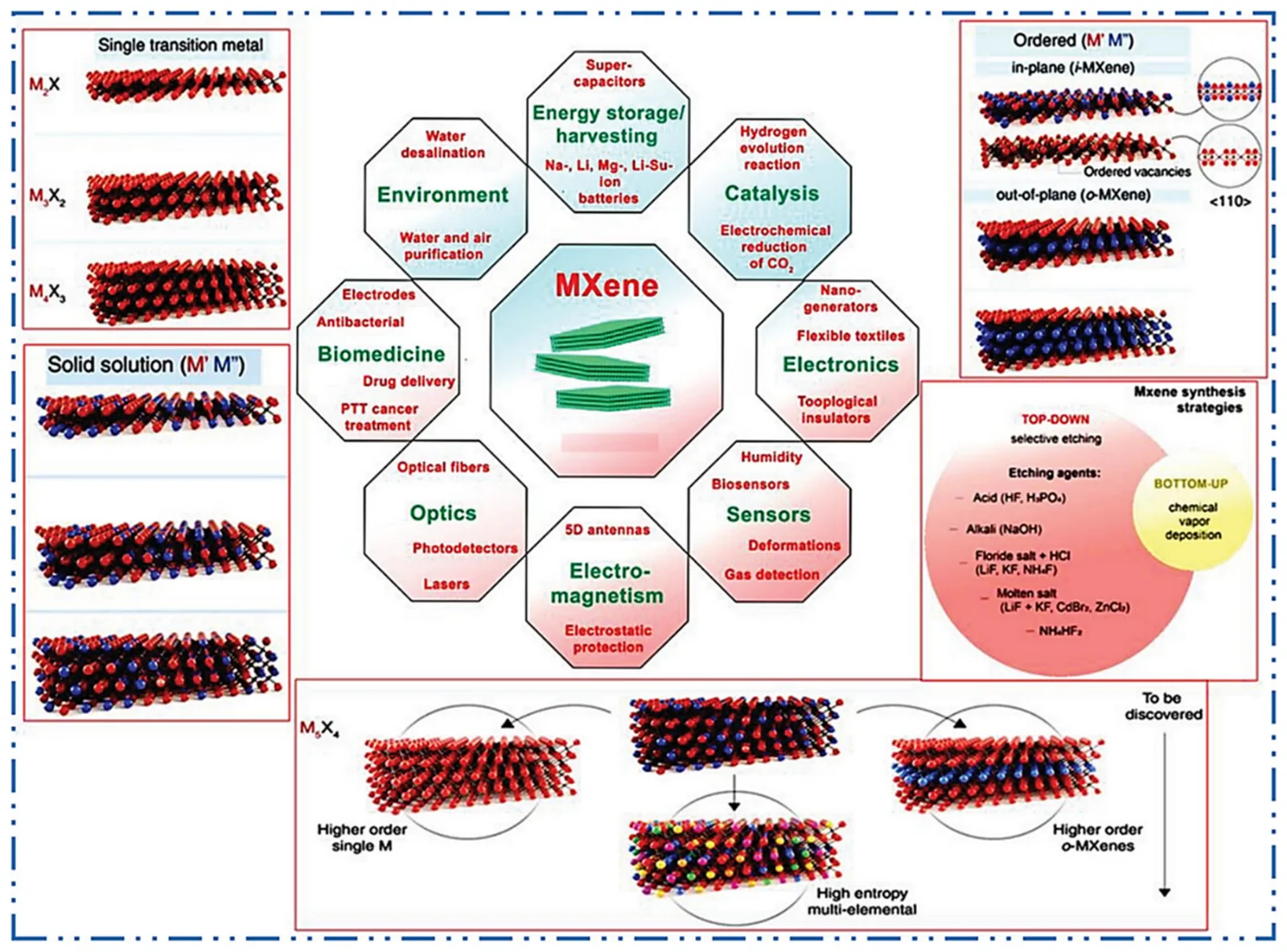
MXene structures, synthesis techniques, and uses are depicted in the scheme. Reprinted from ref. [27].

MXenes are a substantially larger class of two-dimensional transition-metal carbides, nitrides, and carbonitrides that have developed from a mostly Ti-based family. Their overall composition, M_n+1_X_n_T_x_, provides significant options for electrochemical control by enabling independent manipulation of the transition-metal (M), carbon/nitrogen sublattice (X) and surface terminations (T_x_) [17,22,25,26]. This composition flexibility is especially important for the current review because doped non-Ti MXenes provide a logical solution to the drawbacks of pristine electrodes, such as restacking, restricted accessible active sites, slow ion transport, and inadequate pseudocapacitive contribution. The most experimentally developed non-Ti systems, according to recent reviews, are Mo-, V- and Nb-based MXenes. Besides, Cr-, W-, Zr-, Hf-, Ta-, Sc- and Y-based MXenes remain less explored, with several of their features still being mainly supported by theoretical calculations. The various elements of the MAX phases, crystal structure and synthesis of MXenes are shown in Figure 2 [17,22,27,28,29].

### 2.1. Different Non-Ti MXenes

Non-Ti MXenes have attracted increasing attention as a promising class of 2D nanomaterials. Although a lot of research has been done on Ti-based MXenes, investigating MXenes with other transition metals may lead to materials with unique and possibly better qualities. Research on non-Ti MXenes is therefore crucial from both a fundamental and an application-focused standpoint. The special mix of features of non-Ti MXenes is one of the most significant reasons for investigating them. These materials can have remarkable mechanical strength, thermal stability, high electrical conductivity, and intriguing electrochemical and surface properties, depending on their chemical makeup and structure. These features make them appealing for creating cutting-edge functional materials and offer chances to find qualities that might not be possible with traditional Ti-based MXenes. Their wide range of potential uses is another significant feature. Among other uses, non-Ti MXenes are being investigated for energy conversion and storage, catalysis, sensing, electromagnetic interference (EMI) shielding, coatings, and structural materials. Researchers can alter their characteristics for certain uses owing to their 2D structure and customizable chemistry. Thus, new materials and technologies with applications in energy, electronics, environmental protection, and other industries may be developed as a result of ongoing research.

Another key objective for researching non-Ti MXenes is sustainability. Relatively abundant elements and possibly more ecologically friendly material sources could be used to create some non-Ti systems. The creation of substitute materials that can lessen the dependency on particular elements is becoming more and more important as challenges about resource scarcity, environmental effects, and sustainable production continue to rise. Non-Ti MXenes may also provide important technological and financial advantages. Improved energy-storage devices, more effective catalysts, sensitive sensors, sophisticated protective coatings, and lightweight functional materials could all benefit from advancements in these materials. These advancements may foster new technologies and open up new opportunities for the energy, electronics, transportation, and environmental sectors. At the outset, non-Ti MXenes merit significant scientific study due to their varied characteristics, wide range of prospective applications, sustainability opportunities, and potential technical and economic effects. However, elements including composition, surface chemistry, structure, defects, synthesis conditions, and functionalization have a significant impact on the characteristics and functionality of these materials. For the purpose of creating materials with better performance and customizing them for particular uses, it is crucial to comprehend how these parameters regulate how these parameters regulate the characteristics of non-Ti MXenes. The main elements that affect the characteristics and uses of non-Ti MXene nanomaterials are covered in the sections that follow.

#### 2.1.1. Mo-Based MXenes

**Figure 3:**
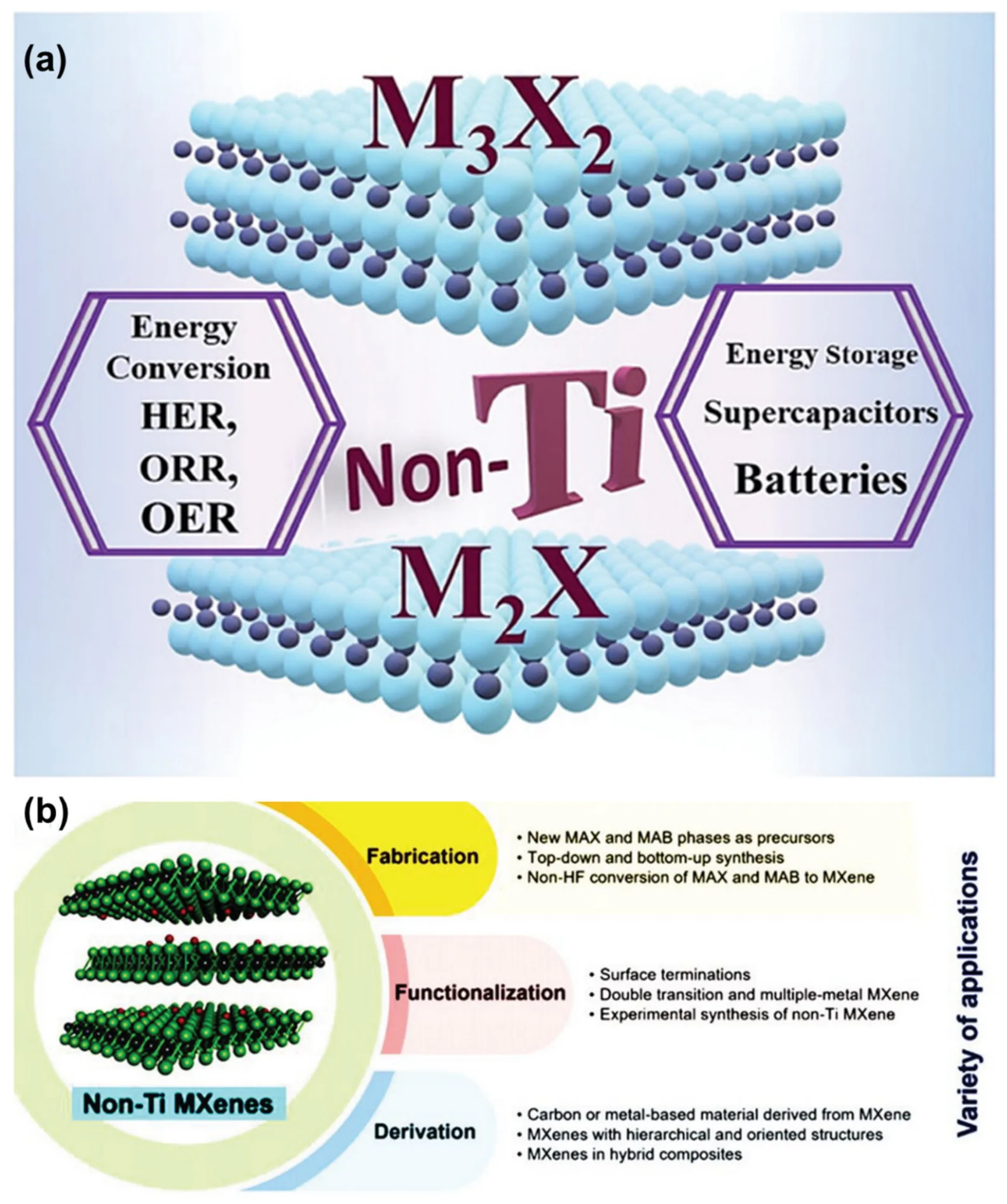
(**a**) Non-Ti MXenes for energy conversion and storage applications and (**b**) Review focus on non-Ti MXenes current and future directions. (**a**) Reprinted from ref. [28] and (**b**) Reprinted from ref. [27].

Particularly, Mo_2_CT_x_ have garnered significant attention among the non-Ti family due to their excellent electrical conductivity, tunable electronic structure, and very strong oxidative resistance. Conventional and fluoride-free etching techniques have been used to generate Mo_2_CT_x_ from Mo_2_Ga_2_C, while bottom-up methods have also been explored, as shown in Figure 3a [23,28,29,30,31]. While emphasizing the fact that surface chemistry and synthesis quality have a significant impact on Mo_2_C electrochemical performance, recent research on the material further validates its promise as a pseudocapacitive electrode [32].

#### 2.1.2. V-Based MXenes

Vanadium can access numerous oxidation states, which makes V_2_CT_x_ and V_4_C_3_T_x_ very appealing for supercapacitors. In addition to MXenes high conductivity and ion-accessible layered structure, this offers intrinsic redox activity [29,33,34]. One of the first non-Ti MXenes to show good supercapacitor performance was V_2_C, which reached a specific capacitance of 487 F/g in 1 M H_2_SO_4_, along with 225 and 184 F/g specific capacitances in MgSO_4_ and KOH, respectively [34]. The electrolyte also influences the dominant charge-storage mechanism. V_2_C exhibited higher capacitance in acidic H_2_SO_4_ than in MgSO_4_ or KOH, reflecting the strong dependence of proton-mediated redox reactions and ion intercalation. Therefore, capacitance values obtained in different electrolytes should not be directly compared without considering the electrolyte-dependent potential window and charge-storage mechanism. Delamination significantly expands the accessible interlayer space, which makes V_4_C_3_T_x_ equally appealing. For instance, after 40,000 cycles, a delaminated V_4_C_3_T_x_ film maintained 93.1% of its initial capacitance and delivered the maximum specific capacitance of 292 F/g [35].

#### 2.1.3. Nb-Based MXenes

Nb_2_CT_x_ and Nb_4_C_3_T_x_, in particular, combine excellent ion accessibility. A large number of surface sites and strong electronic conductivity. More recent research has concentrated on their supercapacitor characteristics [36,37]. Ni inclusion in Nb_2_C boosted the capacitance to 666.67 F/g, which is significant. This rise was attributed to extra electrochemically active sites and a change in the electronic density of states [38]. Similarly, adding Co_3_O_4_ between Nb_2_C layers shortens ion and electron transport channels and suppresses restacking, showing how structural and compositional engineering can improve a non-Ti MXene electrode’s performance [37].

#### 2.1.4. Cr-, W-, Zr-, Hf-, Ta-, Sc- and Y-Based MXenes

The other non-Ti MXenes like Cr-, W-, Zr-, Hf-, Ta-, Sc-, and Y-based MXenes are significant because they broaden the chemical as well as electrical design space. While W-based materials benefit from the electronic structure of a heavy 5d transition metal, Cr-containing MXenes show peculiar electrical and magnetic properties. While Ta_4_C_3_T_x_ has already been shown to function as an electrochemical electrode in experiments, Zr- and Hf-based MXenes are projected to exhibit favorable stability and tunable electronic characteristics [17,22,27,28,29]. In early experiments, Ta_4_C_3_ demonstrated quantifiable supercapacitor activity, demonstrating that heavy-metal MXenes can also take part in electrochemical charge storage [39]. Sc_2_C, Zr_2_C and Y-containing structures have been theoretically investigated for energy-storage applications. Sc- and Y-based systems are still far less explored, as shown in Figure 3b [27,29].

The experimental synthesis of non-Ti MXenes depends on several factors, rather than on the stability of the final MXene structure alone. One of the key factors is the strength of the M-A bond in the MAX precursor; the A-layer must be removed selectively without damaging the M-X framework. The stability and availability of suitable MAX precursors also play an important role in determining whether a particular MXene can be prepared experimentally. In addition, the choice of etchant is critical because insufficient selectivity may lead to incomplete A-layer removal or structural degradation. These factors determine the study of V-, Nb-, and Mo-based MXenes is more extensive, with established experimental routes and characterization data. In contrast, Sc- and Y-based systems remain comparatively less explored, partly because their precursor chemistry and selective etching conditions are less well established. Therefore, the limited experimental reports for these systems are not reported as evidence of inferior properties, but rather as an indication that their synthesis and characterization require more investigations.

### 2.2. Structural Characteristics

Strongly bound M-X layers are divided by an interlayer area with surface terminations, and depending on processing, water, electrolyte ions, or other species make up the basic structure of a non-Ti MXene. When the A element is selectively removed from MAX-type precursors, a conductive M_n+1_X_n_ sheet with exposed transition-metal atoms at the surface is typically left behind [17,22,25]. The identity of M, the M:X ratio, flaws, layer thickness, and surface termination all have a significant impact on the final structure. The electronic structure, work function, wettability, ion adsorption and interlayer spacing can all be significantly altered by -O, -OH, -F and -Cl groups [17,26,40]. After doping, this structural tunability becomes even more crucial. Dopants can locally deform the lattice, redistribute charge, alter the density of states, and produce more electrochemically active centers. They can occupy M, X, vacancy or surface sites [19]. Maintaining the distinction between substitution and doping is particularly important since significant parent element replacement and low-level dopant inclusion can result in quite different structure-property correlations [17]. Doping can simultaneously increase conductivity, reveal active sites, control interlayer spacing, and promote electrolyte penetration. Here, dopants generate defects and expand the interlayer spacing. These effects create a possible consequence of lattice incorporation or structural modification, rather than a general effect of surface termination change.

### 2.3. Charge-Storage Mechanisms in Non-Ti MXenes

The charge-storage behaviour of non-Ti MXenes is best understood as a combination of electric-double-layer capacitance (EDLC), surface pseudocapacitance and interlayer ion-storage processes, rather than a single mechanism [22,25,41,42,43]. Rapid electron transport is made possible by the conductive M-X framework, and electrostatic ion adsorption is made easier by the broad accessible surface. Non-Ti MXenes have a substantial pseudocapacitive contribution due to the rapid reversible redox processes that surface transition-metal atoms and functional groups might experience. In V-based MXenes, pseudocapacitance plays a particularly significant role. Reversible surface redox reactions are made possible by V’s numerous oxidation states, and ion transport is made easier by the comparatively high interlayer spacing. Research on V_4_C_3_T_x_ has demonstrated that ion intercalation and deintercalation take place without catastrophically disrupting the layered structure and that the charge-storage process is primarily surface-controlled [29,35]. The pseudocapacitive reaction of V_4_C_3_T_x_ is significantly influenced by H^+^ intercalation/deintercalation [44]. Nitrogen treatment provides an instructive example of how doping changes this behavior: N-doped V_4_C_3_T_x_ attained 210 F/g, which is higher than the undoped material. This suggests that regulated heteroatom inclusion can enhance the charge-transfer kinetics and electrochemically accessible surface [45].

**Figure 4:**
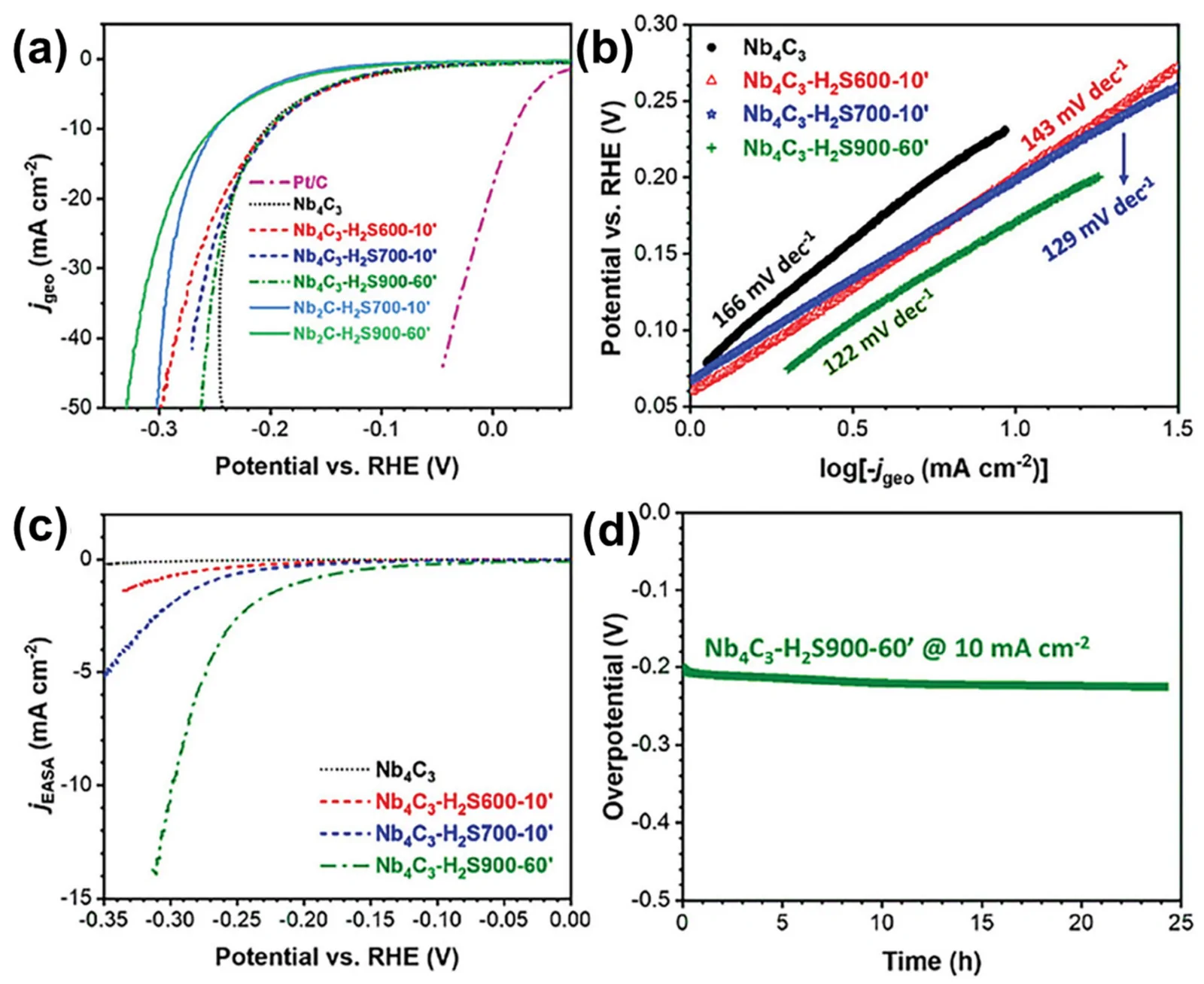
(**a**) The LSC curves for HER were measured at a scan rate of 5 mV/s and adjusted for iR effects, (**b**) The corresponding Tafel slopes for Nb_4_C_3_ pristine, Nb_4_C_3_-H2S600-10′, Nb_4_C_3_-H2S700-10′ and Nb_4_C_3_-H2S900-60′ were determined, (**c**) LSV curves were adjusted for iR drop and normalised to the electrochemically active surface area (EASA) and (**d**) The long-term electrochemical stability of Nb_4_C_3_-H2S900-60′ with a constant current density of 10 mA/cm^2^. Reprinted from ref. [28].

Charge storage in Mo- and Nb-based MXenes reflects the interplay between ion adsorption/intercalation and surface redox chemistry. Both Mo_2_C and V_2_C have large quantum capacitance, according to recent theoretical research, emphasizing the significance of their electronic density of states in dictating the electrochemical response [46]. Ion-surface interactions and the electronic structure can then be changed by surface functionalization and doping. Metal doping has been shown experimentally to alter the density of states in Nb_2_C can directly result in increased capacitance [38]. When the sheets restack, even an intrinsically conductive MXene may lose accessible surface area. The interlayer galleries can be maintained through pillaring, delamination, heterostructure creation, and heteroatom inclusion. Recent V_4_C_3_T_x_/MoO_3_ heterostructures have shown that the designed interface improved pseudocapacitive storage and cyclic stability [47]. More recently, Ta_4_C_3_T_x_ pillared with N-doped graphene quantum dots showed a seven-fold improvement in capacitance when compared to pure Ta_4_C_3_T_x_, demonstrating the powerful impact of interlayer engineering on a heavy-metal MXene [48]. Therefore, the main benefit of supercapacitors goes beyond just their large surface area. The combination of metallic conductivity, ion adsorption, accessible surface redox chemistry, and quick interlayer transport is the strength. By altering the electrical structure and generating new active sites without necessarily destroying the original 2D framework, doping gives an additional degree of control, as shown in Figure 4a–d [28]. Doped Cr-, W-, Zr-, Hf-, Sc- and Y-based systems constitute an expanding research area, whereas doped Mo-, V-, Nb- and Ta-based MXenes are particularly promising. However, recent reviews highlight the fact that many of these latter compositions are still primarily theoretical and that issues with controlled termination, scalable synthesis, air stability and reproducible M-site doping still need to be addressed [17,22,27,28,29]. Charge storage in non-Ti MXenes involves a combination of EDLC, surface pseudocapacitance and interlayer ion storage, with their relative contributions depending on composition and surface chemistry. Their kinetics can be evaluated using: i = aV^b^, where b approaching 1 indicates surface-controlled behavior, while b near 0.5 represents the ideal diffusion-limited case. Dunn’s method, (i(V) = k_2_*v* + k_2_*v*^1/2^), further separates capacitive and diffusion-limited dependent contributions. These analyses provide a rate capability rather than capacitance values. For example, N-doped Nb_2_CT_x_ increased the capacitive contribution from 31.1% to 64.64%, while lowering charge-transfer resistance can lead to faster and more accessible charge-storage pathways. Chen et al. [49] have reported that N/O/P co-doped hollow carbon nanofibers improved ion/electron transport and accelerated polysulfide conversion through chemisorption and catalytic effects.

## 3. Doping Strategies for Different Non-Ti MXenes

Doping has grown in importance as a method for modifying the electrochemical behavior of non-Ti MXenes because the parent materials frequently have to choose between high electrical conductivity, accessible surface area and enough redox activity, doping has grown in importance as a method for modifying the electrochemical behavior of non-Ti MXenes. Controlled doping can alter the MXene lattice, electronic structure, interlayer environment, and surface chemistry at the atomic level instead of just introducing another active material. M-site replacement, X-site heteroatom doping, T-site/surface modification and in-situ or ex-situ methods are the main categories into which recent research has divided MXene doping [17,19,40,41]. Lattice doping is distinguished from surface modification and the formation of separate phases. M-site doping refers specifically to substitution/replacement of host metal atoms within the MXene lattice, whereas X-site doping involves incorporation of heteroatoms into the C/N sublattice. T-site modification refers primarily to changes in surface terminations or functional groups. In contrast, surface-deposited metals, nanoclusters, quantum dots used for pillaring, and secondary oxide or fluoride phases constitute separate structural components rather than direct evidence of lattice substitution. For Mo-, V-, Nb-, Ta-, and other non-Ti MXenes meant for high-rate supercapacitor electrodes, these approaches are particularly significant.

### 3.1. M-Site Doping and Transition-Metal Substitution

**Figure 5:**
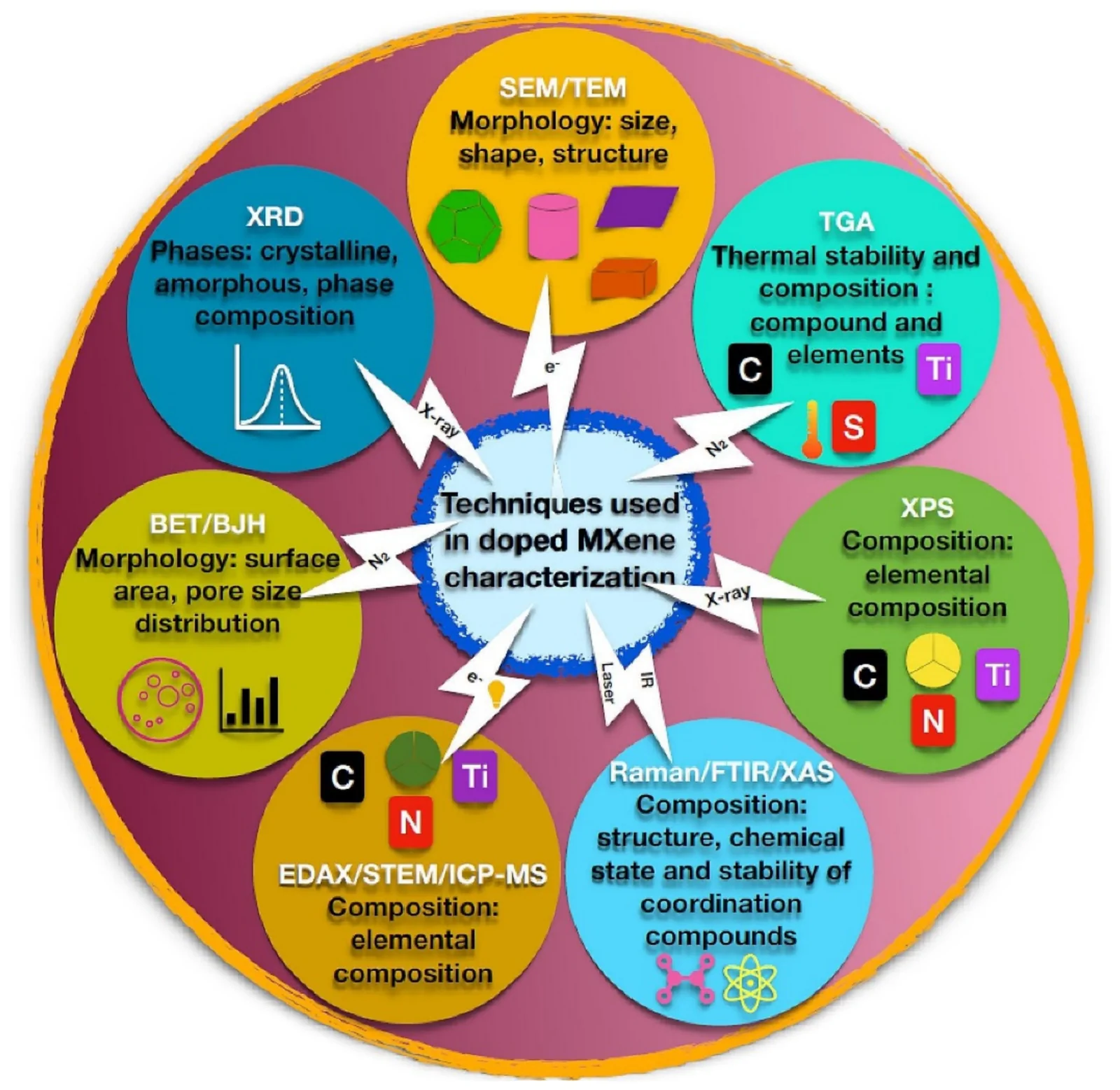
An overview of doped MXenes characterization methods. Reprinted from ref. [19].

M-site doping is substituting a different metal for a portion of the transition-metal atoms in the MXene lattice. This strategy is appealing since the M site directly controls the material’s electronic structure and M-X bonding. Charge can be redistributed, and the density of states close to the Fermi level can be altered by substituting metals with various electronegativities, atomic radii or oxidation states [17,23,40]. For instance, it has been shown that transition-metal alteration of Nb_2_C can effectively alter its electronic structure and increase the number of electrochemically accessible sites. For example, compared to pristine Nb_2_C, Ni-modified Nb_2_C demonstrated an improved electrochemical performance [38]. This approach is especially intriguing for non-Ti MXenes because it combines the inherent characteristics of two distinct transition metals. Combinations of V-Mo, V-Nb, Mo-W, or Nb-Ta, for instance, may be able to balance electrical conductivity with redox activity. However, it is necessary to carefully distinguish between the production of various metal-containing phases and actual lattice replacement using methods like XPS, XRD, STEM-EDS, and XAS. Uncontrolled replacement can readily lead to heterostructure or composite creation instead of accurate atomic doping. An overview of doped non-Ti MXenes characterization techniques is illustrated in Figure 5 [17,19]. However, the presence of a dopant element alone does not establish M-site substitution. Metal deposition, nanocluster formation, or secondary-phase growth can produce similar compositional and spectroscopic signatures without incorporation into the MXene lattice. Therefore, confirmation of true lattice substitution requires evidence of the dopant position and local coordination, preferably through HAADF-STEM combined with EDS/EELS and complementary X-ray absorption spectroscopy (XANES/EXAFS). Conventional XPS and XRD are useful for identifying chemical states and crystalline phases but are generally insufficient by themselves to establish site-specific atomic incorporation.

### 3.2. X-Site Heteroatom Doping

Heteroatoms like N, S, P, B, or O are integrated into or have significant interactions with the C/N sublattice in X-site doping. Because N can alter the electronic structure while adding more electrochemically active sites, nitrogen doping has drawn a lot of attention for supercapacitors. Three potential N-doping configurations distinguished in recent literature are surface adsorption, lattice substitution and functional group substitution [50]. Conductivity is not the only consequence. Defects, changes in the charge distribution surrounding transition-metal atoms, ion adsorption energies, and occasionally an increase in interlayer distance can all originate from heteroatom inclusion. These modifications may enhance the contribution from surface-controlled pseudocapacitance and improve electrolyte-ion transfer. In addition to emphasizing CVD, solution-based treatment and multi-doping as new methods, a recent study from 2026 explicitly highlighted N, S and P doping as efficient ways to alter MXene interlayer spacing, ion diffusion and charge-storage behavior [51]. Inclusion of N is especially intriguing for V-based MXenes because it can enhance the balance between conductivity and pseudocapacitance and complement the intrinsic multivalent redox chemistry of vanadium.

### 3.3. T-Site/Surface Functional-Group Modification

T-site modification concentrates on the surface termination chemistry, in contrast to traditional lattice doping. Depending on the precursor and etching circumstances, pristine MXenes typically contain combinations of -O, -OH, -F, and occasionally -Cl groups. Ion adsorption, wettability, electronic structure and electrochemical stability are all significantly impacted by these groups [17,19]. Therefore, the electrode/electrolyte interface can be adjusted without significantly disrupting the M-X framework by substituting or altering these groups with species that contain O, S, N, Se, or P [17,40]. This method is particularly useful for non-Ti MXenes that are structurally sensitive or challenging to synthesise. For instance, O-rich terminations can promote aqueous-electrolyte interactions, while surface functionalization of Mo_2_C and Nb_2_C can change ion-binding strength and charge transfer kinetics. Surface termination is no longer treated as a synthesis by-product but rather as an active design parameter in recent studies [17,51]. This distinction is crucial for supercapacitors because a large portion of the quick pseudocapacitive reaction starts at the surface, where electrolyte ions initially contact the electrode.

### 3.4. In-Situ and Ex-Situ Doping Approaches

Doping can be broadly classified as either in-situ or ex-situ from the standpoint of synthesis. In order to incorporate the dopant into the lattice prior to selective etching, in-situ doping inserts the desired element during precursor/MAX-phase synthesis. This method is especially useful for M- or X-site replacement and can produce a suitable homogeneous atomic distribution [28,41]. In contrast, ex-situ doping is carried out following the creation of MXene using techniques including hydrothermal/solvothermal treatment, annealing, plasma processing, CVD, or solution-based processes. Since the dopant concentration and surface chemistry can be changed after the 2D structure has formed, these techniques are typically more adaptable [41,50]. The location of the dopants’ intended residence ultimately determines which of the two approaches is optimal. While ex-situ treatment provides more control over surface functionalization and near-surface doping, in-situ synthesis is more appropriate for complete lattice substitution [41]. According to recent findings, it may be possible to more successfully independently adjust conductivity, ion transport and pseudocapacitance by combining the two strategies, such as lattice substitution followed by surface heteroatom modification [28,51]. Therefore, a single type of doping is unlikely to be the most promising approach for doped non-Ti MXenes. Rather, the electrical structure and electrode/electrolyte interface may be tuned concurrently by logical combinations of M-site replacement, X-site heteroatom insertion, and T-site engineering. Achieving uniform dopant distribution without rupturing the multilayer MXene structure is the main obstacle. As a result, future research should go beyond just reporting increased capacitance and establish precise structure-composition property connections that clarify why a specific dopant enhances supercapacitor performance.

## 4. Doping-Induced Structure-Property Relationship for Non-Ti MXenes

Doping is more than just changing the composition of non-Ti MXenes; doping offers a method to consciously connect atomic structure to electrochemical function. An adequately chosen dopant can create defects, open interlayer diffusion channels after surface terminations, accelerate charge-transfer events, and redistribute electronic charge in Mo-, V-, Nb-, Ta-, and other non-Ti MXenes. These effects are especially significant for supercapacitors, because quick ion accessibility, reversible surface redox and low interfacial resistance must all happen at the same time, as high conductivity alone is insufficient [17,19,22,27,40]. Recent studies emphasize that the electrochemical benefit of doping depends not only on the concentration of the dopant but also on its position, bonding arrangement and interaction with the electrolyte [23,28,41]. Doping can produce substantial changes in the supercapacitor behavior of non-Ti MXenes because even a small change in composition can modify several properties simultaneously. Dopant atoms can redistribute electronic charge, alter the density of states, generate defects or additional electroactive sites, and modify the interlayer spacing and surface terminations. These changes can facilitate electrolyte-ion access and diffusion, improve charge-transfer kinetics, and increase the contribution from reversible surface redox reactions. Consequently, doping can influence not only the specific capacitance but also the rate capability, energy-storage efficiency, and cyclic stability.

### 4.1. Electronic-Structure and Charge-Distribution Engineering

Modification of the electronic structure and local charge distribution is the initial and possibly most fundamental effect of doping. In many MXenes, the electronic states surrounding the Fermi level are dominated by transition-metal d-states. As a result, the Fermi level can be shifted, the density of states can be changed, and the intensity of ion-surface interactions can be altered by introducing heteroatoms or replacing a portion of the host atoms [33,52]. This is especially relevant for MXenes based on V-, Mo-, and Nb-, where significant pseudocapacitive activity is already provided by the transition-metal electronic structure. Because the dopant and host atoms typically have different electronegativities and valence states, M-site replacement can result in local charge polarization. Such redistribution may produce more redox-active states and reduce the energy needed for charge transfer between the electrolyte and electrode. For instance, recent theoretical research on Sc-based MXenes shows that metal-layer intercalation can significantly alter quantum capacitance and charge-storage properties, demonstrating how modifications in the electronic environment translate into electrochemical behaviour [53]. Another method is heteroatom doping, where N, P, S, and B can simultaneously affect ion adsorption and change the electron density surrounding nearby transition-metal atoms [23,50,51].

### 4.2. Defects, Vacancies, and Active-Site Generation

In doped MXenes, defects should not always be considered as structural flaws. They can develop into electrochemically beneficial active sites with appropriate supervision. Additional transition-metal atoms are exposed, their coordination environment is altered, and the electronic structure is locally distributed by metal vacancies, carbon vacancies and substitutional defects. The kind, density and distribution of vacancies can control electrical characteristics, ion-diffusion barriers, surface chemistry and capacitive behavior as per a review of vacancy-engineered MXenes [37]. This is especially helpful for supercapacitors since a vacancy can allow electrolyte ions to reach a metal core that would otherwise be unreachable. Surface redox processes may be facilitated and ion adsorption strengthened by the newly exposed locations. Simultaneously, the conductive M-X network may be disrupted, and the layered structure may become unstable due to excessive vacancy development. Therefore, an ideal defect concentration is necessary, and it does not imply that having more defects means better performance of the material. Similar findings from recent defect-engineering research indicate that while uncontrolled defects can become transport obstacles, regulated carbon vacancies can produce more redox-active sites and enhance ion-storage kinetics [54]. Defects and vacancies can enhance the performance of supercapacitors by introducing additional electrochemically accessible sites and modifying the electronic structure of the MXene surface. These sites can facilitate electrolyte-ion adsorption and reversible surface redox reactions. Moreover, controlled defects can reduce the ion-transportation pathways and improve charge-transfer kinetics. However, excessive defects may disrupt the conducting framework of MXenes and accelerate structural degradation. Thus, the electrochemical benefit depends on the type, concentration, and distribution of defects rather than simply on increasing defect density.

### 4.3. Interlayer-Spacing and Ion-Transport Regulation

The interlayer space is one of the most critical structural factors influencing the rate capability of MXene electrodes. When individual nanosheets are restacked, the accessible surface is decreased, and electrolyte ions are forced through convoluted pathways. Doping can solve the issue by introducing bigger atoms, creating vacancies, causing local lattice distortion or forming chemical interactions that function as nanoscale spacers [55,56,57]. Although excessive expansion may lower volumetric capacitance or weaken electrical connection between adjacent layers, a bigger and more accessible interlayer distance often lowers the barrier for ion movement. Ion size alone does not dictate accessibility. According to recent ion-transport research, the hydration state, solvent interactions, surface termination and interlayer charge also regulate the effective diffusion process [55]. Because the quickest electrodes must offer both continuous electron-conduction networks and short ion-transport channels, this balance is particularly crucial for supercapacitors. According to recent structural engineering research, one of the main obstacles for MXene supercapacitors is precisely this trade-off between high volumetric capacitance and quick ion transport [57].

### 4.4. Surface Chemistry, Wettability, and Redox Activity

Another benefit is that the doping modifies the MXene/electrolyte interface chemistry. Hydrophilicity, ion adsorption, surface charge and the presence of redox-active sites are all determined by surface terminations like -O, -OH, -F and -Cl [25,26,29]. These groups can be changed or replaced by a dopant, which can also change the strength of their binding or produce novel functional configurations. How effectively an aqueous electrolyte penetrates the electrode can be determined by the ensuing changes in wettability. For MXenes based on V and Mo, where surface transition-metal sites can directly contribute to pseudocapacitance, this effect is particularly significant. According to recent research, the Mo_2_C layered structure, having metallic conductivity and adjustable surface chemistry, contributes significantly to its supercapacitor performance [32]. Surface termination needs to be improved rather than just maximized, as new studies on nonfunctionalized MXenes also indicate that traditional surface groups may occasionally impede conductivity and electrochemical performance [58].

### 4.5. Electrical Conductivity and Electrochemical Kinetics

The structural changes produced by doping must translate into faster electron and ion transport. A successful dopant should reduce charge-transfer resistance and ion diffusion while simultaneously increasing electronic conductivity or maintaining the highly conductive MXene framework. These changes are often captured by electrochemical impedance spectroscopy through decreases in diffusion-related resistance and charge-transfer resistance. Heteroatom-doped MXenes clearly demonstrate the synergistic nature of this connection. For instance, N doping can simultaneously improve electrical transport, increase active-site accessibility, alter surface wettability and induce defects [51,55]. Similar to this, recent nitrogen-doped MXene nanomeshes show how atomic alteration in conjunction with porous architecture can produce new ion-transfer channels and significantly enhance the performance of both volumetric and gravimetric supercapacitors [59]. Overall, rather than a single electrical impact, the improved performance of doped non-Ti MXenes can be explained by a number of interrelated structural and chemical modifications. Electrolyte-ion transfer can be facilitated by the introduction of a dopant, which can also alter the interlayer structure, produce defects and extra active sites and redistribute the charge within the MXene framework. In addition, alterations in surface chemistry and wettability can enhance the electrode-electrolyte interaction, facilitating quicker ion and electron transfer. Both electric double layer capacitance (EDLC) and pseudocapacitive charge storage can be enhanced by these combined actions. Therefore, rather than only comparing individual capacitance values, it is more instructive to comprehend the interaction between the dopant, its concentration, location, and the resulting structural changes for doped non-Ti MXenes. At the outset, this review can direct the logical development of MXene electrodes with enhanced rate capability, energy-storage efficiency, and long-term stability. Heteroatom doping can improve electrochemical kinetics by changing both the electronic structure and the pathways available for ion transport. N, S, P, B, and O dopants can modify surface charge, defect density, wettability, and interlayer spacing, making active sites more accessible to electrolyte ions. The kinetic effect is calculated using i = aV^b^ and Dunn’s analysis by: i(V) = k_2_*v* + k_2_*v*^1/2^. A larger surface-controlled contribution at higher scan rates generally indicates improved ion accessibility and faster charge transfer. In N-doped Nb_2_CT_x_, the capacitive contribution increased from 31.1% to 64.64% due to lower charge-transfer resistance. Li et al. [60] have demonstrated that hollow hard-carbon nanofibers provided larger interfacial contact, shorter transport pathways, and lower interfacial and diffusion resistance than solid fibers.

In general, the changes in the electronic structure, defect chemistry, and interlayer spacing have been supported by experimental characterization in some non-Ti MXenes, while other mechanistic explanations remain primarily theoretical. We have therefore distinguished experimentally observed effects from computational predictions throughout this section and identified areas requiring further experimental validation.

## 5. Supercapacitor Applications of Doped Non-Ti MXenes

The electrochemical behavior of non-Ti MXenes can be significantly changed by introducing foreign elements into their lattice or surface. Doping has emerged as a useful strategy for overcoming some of the limitations of pristine non-Ti MXenes, particularly limited active-site density, restacking, sluggish ion transportation, and inadequate control over electronic structure. Unlike pristine non-Ti MXenes, doping provides additional contributions towards the electronic states, surface chemistry, defect density, and ion–accessible sites that govern charge storage [61,62,63,64,65]. Recent studies have explored both heteroatom and transition–metal incorporation. These modifications have produced notable improvements in specific capacitance, rate capability, and cyclic stability, although the improvement varies with the dopant, host MXene, doping level, electrolyte, and electrode architecture. Importantly, some of the highest reported performances arise from combining doping with structural or interfacial engineering, making it difficult to separate the contribution of the dopant alone. This section therefore focuses on experimentally demonstrated doped non-Ti MXenes and critically examines how dopant element, incorporation strategy, structural modification, and electrochemical response are interconnected.

**Figure 6:**
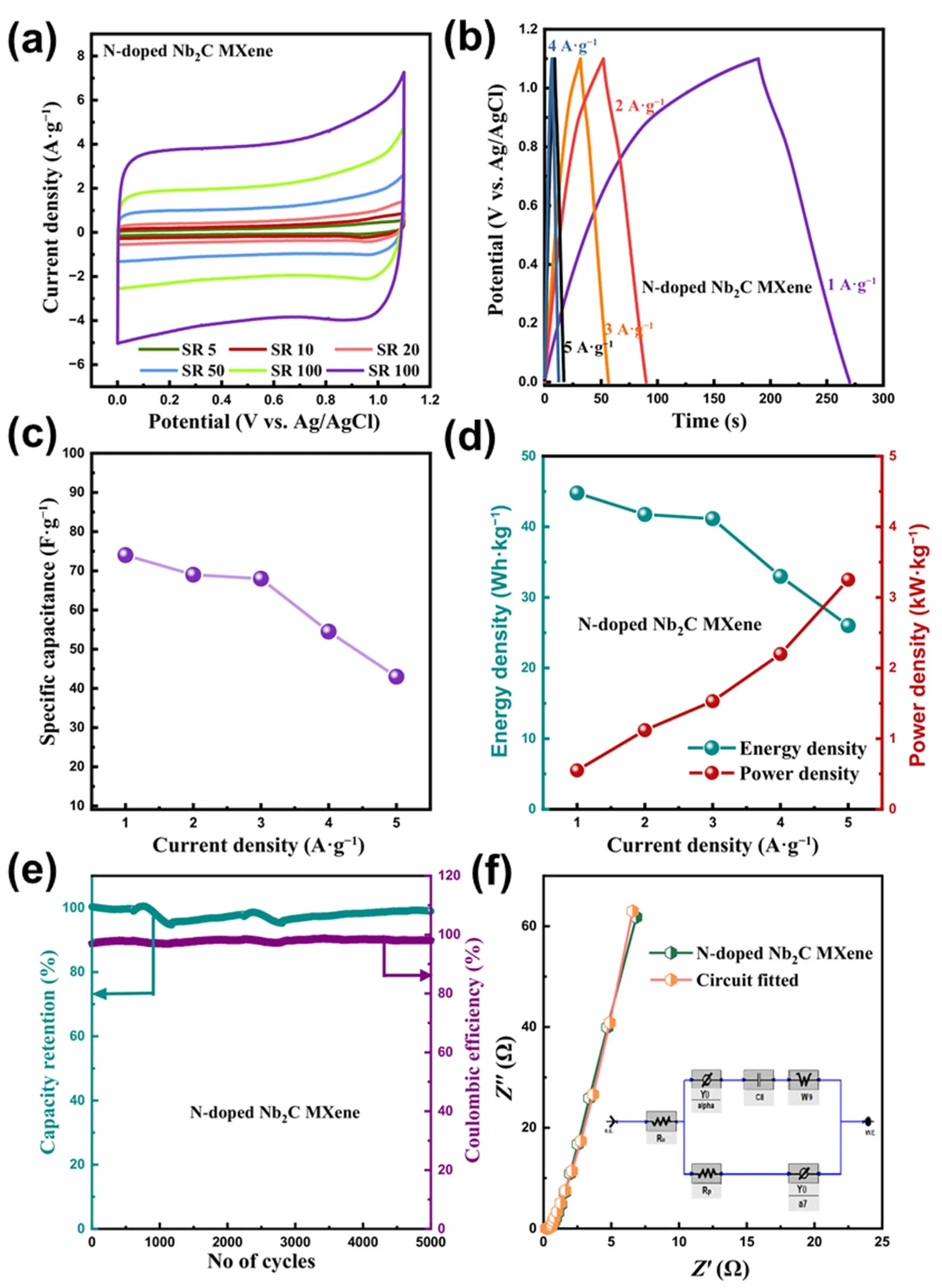
Electrochemical performances of the N-Nb_2_CT_x_//AC asymmetric supercapacitor device: (**a**) CV plots at different scan rates, (**b**) GCD analysis at different current densities, (**c**) Current density vs. specific capacitance plot, (**d**) Ragone plot of the device, (**e**) Coulombic efficiency and cyclic stability up to 5000 cycles at 5 A/g current density, and (**f**) Fitted Nyquist plot. Reprinted from ref. [67].

Among non-Ti MXenes, N doping provides the effect of heteroatom doping on supercapacitor performance. Li et al. introduced N into V_4_C_3_T_x_ by annealing the MXene in NH_3_ [45]. The optimized NH_3_-treated sample prepared at 350 °C delivered a specific capacitance of 210 F/g at 10 mV/s in 1 M H_2_SO_4_ in a three-electrode configuration. The electrode also showed 96.3% capacitance retention after 10,000 GCD cycles at 10 A/g. The improvement is associated with the incorporation of N and the simultaneous modification of the surface chemistry. NH_3_ treatment removes part of the F-containing surface species and changes the local electronic environment of V_4_C_3_T_x_. The optimized doping level improves conductivity and electrochemical accessibility. The observed improvement in capacitance and diffusion behavior may therefore result from the combined effects of N incorporation, altered surface terminations, and changes in the defect structure. The decent cyclic stability also indicates that moderate N incorporation does not severely compromise the structural integrity of the V_4_C_3_T_x_ layers. The performance depends strongly on the doping condition. The 350 °C sample performed better than the sample treated at a higher temperature, showing that excessive modification can alter the MXene structure and surface chemistry negatively. Therefore, dopant concentration and thermal treatment are important design variables, rather than secondary synthesis parameters. A more advanced strategy was demonstrated by Nasrin et al., who reported the materialization of N-doped Nb_2_CT_x_ and reduced graphene oxide to form a (N-Nb_2_CT_x_/rGO) 2D/2D heterostructure [66]. This approach is important because it combines electronic doping with interfacial and structural engineering rather than relying on doping alone. The material delivered a specific capacitance of 816 F/g at 1 A/g in a three-electrode configuration. The symmetric supercapacitor device using N-Nb_2_CT_x_/rGO electrode achieved an energy density of 29 W h/kg in aqueous H_2_SO_4_ and 33 W h/kg in TEABF_4_/ACN. The aqueous device also reached a reported power density of approximately 6000 W/kg. Most notably, the quasi-solid-state device retained 100% of its initial capacitance after 100,000 charge–discharge cycles. A study reported N-functionalized Nb_2_CT_x_ prepared using a hydrothermal process [67]. The N-doped Nb_2_CT_x_ MXene delivered a specific capacitance of 640 F/g at 5 mV/s, compared with 276 F/g for pristine Nb_2_CT_x_ in a three-electrode study. An asymmetric supercapacitor device of N-Nb_2_CT_x_//activated carbon (AC) was fabricated for real-time applications. The electrochemical performance of the device is shown in Figure 6. CV curves of the asymmetric device indicate pseudocapacitive behavior (Figure 6a) with a specific capacitance of 75 F/g at 1 A/g (Figure 6b,c). The Ragone plot of the device shows that at the power delivery rate of 0.55 kW/kg, it retained an energy density of 45 W h/kg (Figure 6d). The asymmetric supercapacitor device of N-Nb_2_CT_x_//AC retained 90% of its capacitance after 5000 cycles at 5 A/g (Figure 6e) with low resistance for charge transfer, as evidenced by the Nyquist plot (Figure 6f). The improvement was associated with N-induced modification of surface terminations, particularly the replacement of F-containing species, together with increased pseudocapacitive activity. DFT calculations further indicated that the N-modified structure is thermodynamically favorable. Doping has emerged as an effective way to overcome the restacking and limited ion–accessibility of non-Ti MXenes [68]. A recent example is N-functionalized Nb_2_CT_x_, where N incorporation expands the interlayer spacing, creates additional electroactive sites, and improves charge transport [68]. The synthesis strategy for N-Nb_2_CT_x_ MXene includes selective etching and N functionalization. The optimized N-Nb_2_CT_x_ treated at 400 °C delivered a specific capacitance of 382 F/g at 1 A/g, compared with only 150 F/g for pristine Nb_2_CT_x_. More importantly, its capacitance remained 140 F/g at 20 A/g, corresponding to 36.8% retention, whereas the undoped MXene retained only 27 F/g (18.14%). This improvement is linked to the formation of Nb–N bonds, suppressed nanosheet restacking, and a higher contribution from surface-controlled charge storage. At 10 mV/s, the capacitive contribution increased from 31.1% for Nb_2_CT_x_ to 64.64% after N doping, while the charge-transfer resistance decreased from 1.43 to 1.20 Ω. In the assembled N-Nb_2_CT_x_//AC hybrid supercapacitor, the device achieved 23.36 W h/kg at 900 W/kg and retained 2.56 W h/kg at 1.7 kW/kg power density. It also maintained 92.31% of its capacitance after 12,000 cycles with 98.63% Coulombic efficiency. These results show that doping can simultaneously improve ion accessibility, conductivity, and structural stability. However, excessive doping or high-temperature treatment may promote oxidation and reduce performance, indicating that dopant concentration and processing conditions must be carefully controlled. Zhu et al. developed structurally stable N-doped Mo_2_C/C nanobelts with a 1D tunnel-like structure [69]. The electrode showed an initial capacitance of 1139 C/g at 1 mV/s, 151 C/g at 2000 mV/s, and 208 C/g at 200 A/g. After electrochemical activation, the capacitance increased to 2523 C/g at 1 mV/s and 1403 C/g at 50 A/g after 15,000 cycles. The authors attributed the excellent behavior to the combination of N doping, the intrinsic 1D tunnel structure of Mo_2_C, and carbon stabilization. More than 90% of the charge storage was surface-controlled above 10 mV/s, indicating rapid kinetics. These results are important because it shows that N doping can be effective for V-, Mo-, and Nb-based MXenes, suggesting that the benefit is not limited to a particular transition–metal host.

**Figure 7:**
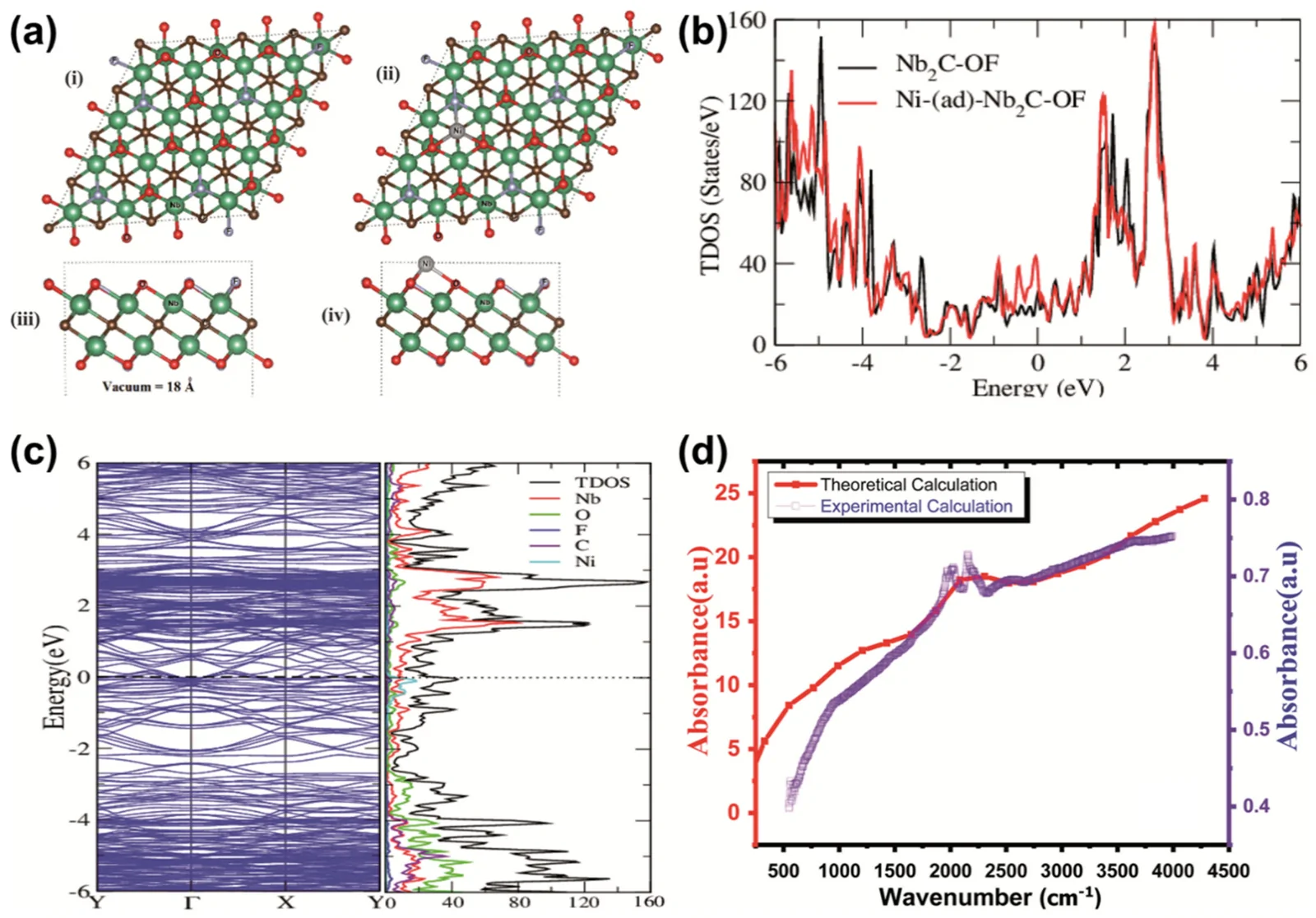
(**a**) DFT-optimized structure of Nb_2_C–OF MXene and Ni-adsorbed Nb_2_C–OF MXene, (**b**) Total density of states (TDOS) for Nb_2_C–OF and Ni–adsorbed Nb_2_C–OF, (**c**) Band structure and orbital-resolved density of states (DOS) of Nb_2_C–OF MXene and Ni-adsorbed Nb_2_C–OF MXene, and (**d**) Experimental and theoretical optical properties of doped MXene. Reprinted from ref. [38].

Metal doping provides a different approach to improve the electrochemical performance of non-Ti MXenes. Zaheer et al. reported the Ni-doped Nb_2_C MXene for supercapacitor applications [38]. The DFT-optimized structures confirm stable Ni adsorption on Nb_2_C, indicating strong interaction between Ni and the MXene surface (Figure 7a). The pristine Nb_2_C electrode delivered a specific capacitance of 258.6 F/g at 5 mV/s, whereas Ni-doped Nb_2_C reached 666.67 F/g capacitance under the same measurement conditions in PVA–H_2_SO_4_ electrolyte. Thus, Ni incorporation increased the measured capacitance by approximately 2.6 times. The doped electrode retained about 81% of its initial capacitance after 10,000 cycles. The enhancement is mainly due to the changes in the electronic structure and the generation of additional active sites. DFT analysis showed an increase in the density of states near the Fermi level after Ni incorporation, which can facilitate electronic transport (Figure 7b). The band structure, as shown in Figure 7c, shows a nearly zero bandgap and metallic behavior, with Nb and Ni states crossing the Fermi level. The DOS also indicates contributions from Nb 4d, Ni 3d, and C 2p orbitals, confirming strong orbital hybridization. The calculated optical absorption profile closely matches the experimental spectrum, supporting the reliability of the theoretical model and confirming the modified optical response of Ni-doped Nb_2_C (Figure 7d). Experimentally, Ni doping also increased the electrochemical response compared with pristine Nb_2_C. This result demonstrates an important advantage of M-site or metal doping. Unlike N doping, which mainly modifies the anion or surface environment, incorporation of a transition metal can directly modify the metal-centered electronic states responsible for charge transfer and surface redox reactions. However, the 81% retention after 10,000 cycles is lower than the stability reported for N-doped V_4_C_3_T_x_ and N-Nb_2_CT_x_/rGO 2D/2D heterostructure. Therefore, the increase in capacitance reduces long-term retention. A particularly interesting study reported post-synthetic La incorporation into Nb_2_CT_x_ and Nb_4_C_3_T_x_ [70]. This study represents an experimentally demonstrated metal-incorporation strategy beyond conventional N doping. DFT and experimental characterization indicated favourable La incorporation together with La_2_O_3_ and LaF_3_ formation, which modified the surface chemistry and charge-transfer behavior. La-incorporated Nb_2_CT_x_ delivered an areal capacitance of 200.5 mF/cm^2^ at 5 mV/s within a 1.5 V window, together with an energy density of 0.47 mWh/cm^2^ and power density of 937.3 mW/cm^2^ over 6000 cycles. More remarkably, La-incorporated Nb_4_C_3_T_x_ showed a 291% increase in capacitance after 90,000 cycles, though with a lower coulombic efficiency. La incorporation effectively addresses the limited electrochemical activity and conductivity of Nb-based MXenes by modifying their structure, surface chemistry, and charge-storage mechanism. In Nb-based MXenes, La formed La–Nb and La–C bonds and additional La_2_O_3_ and LaF_3_ phases, which increased interlayer spacing, facilitated ion transport, and provided additional redox-active sites. However, the unusually high retention of La-incorporated Nb_4_C_3_T_x_ should be interpreted because its coulombic efficiency decreased to 30%, indicating contributions from parallel reactions and possible water electrolysis. Overall, La improves charge transfer, ion accessibility, redox activity, and structural stability, but optimizing La content and suppressing side reactions remains essential for reliable practical performance.

The available results nevertheless show that doping can produce substantial changes in supercapacitor behavior. The main effects arise from modified electronic states, additional redox-active sites, increased interlayer spacing, altered surface termination, and improved electrolyte accessibility. Importantly, the improvement does not always originate from a larger surface area. In some cases, changes in electronic structure and surface chemistry contribute more strongly to capacitance than the geometric surface area. This distinction is important when assessing the actual role of doping. At present, N doping has the strongest direct experimental evidence among non-Ti MXenes for supercapacitors, while metal doping offers a promising route for stronger electronic-structure modulation. Capacitance enhancement after doping should not be attributed solely to the dopant without considering the structural changes introduced during the doping process. Dopant incorporation may cause the formation of defects or vacancies, changes in surface termination, altered wettability, and modification of interlayer spacing. These changes can independently affect ion accessibility, surface redox activity, and charge-transfer kinetics. The distinction should be maintained because the enhancement may arise from a secondary phase rather than from the dopant itself. The electrochemical energy storage performances of some non-Ti MXene-based electrodes for supercapacitor application are summarized in Table 1. Active mass loading is another important factor when assessing the practical performance of doped non-Ti MXene electrodes. Lower mass loading can favor electrolyte accessibility and ion transport. Increasing the mass loading of active materials may enhance the diffusion resistance and can trigger nanosheet restacking, thereby reducing the utilization of electrochemically active sites. At the same time, high packing density can be beneficial for volumetric energy storage. Therefore, gravimetric capacitance should be considered with mass loading. The limited reports of these parameters in the existing literature also prevent a reliable comparison of the practical volumetric performance of doped non-Ti MXene electrodes.

However, doped non-Ti MXenes have demonstrated a clear potential to improve supercapacitor performance, but the experimental database is still small. N-V_4_C_3_T_x_, Ni-Nb_2_C, and N-Nb_2_CT_x_/rGO provide the strongest direct examples, showing improvements in capacitance, energy density, rate capability, and cyclic stability. The next step should be to move from empirical doping toward site-controlled and concentration–controlled doping, supported by spectroscopy, DFT, and standardized electrochemical testing. Such an approach will be essential for establishing a reliable dopant–structure–charge-storage relationship and for identifying which non-Ti MXenes can deliver genuine advantages at the full-device level. The available experimental research on doped non-Ti MXenes is still limited and is focused mainly on V-, Nb-, and Mo-based systems. The electrochemical response varies with the dopant, host MXene, dopant concentration, electrolyte, and electrode architecture. Further studies using different dopants and host structures under comparable testing conditions are needed to establish reliable structure–performance relationships.

## 6. Structure Performance Relationship of Doped Non-Ti MXenes in Supercapacitors

The examples discussed in Section 5 show that doping can improve the electrochemical response of non-Ti MXenes, but the improvement is not controlled by dopant concentration alone. The same dopant can produce different results when its concentration, location, bonding state, or processing temperature changes [45,66,68]. In the N–doped Nb_2_CT_x_ system, increasing the treatment temperature to an optimum level increased both the capacitive contribution and rate capability [68]. These results indicate that the performance of doped non-Ti MXenes should be discussed through a connected structure–doping–mechanism–performance framework rather than by comparing capacitance values only. The following discussion therefore correlates how the nature of the dopant and concentration influence structure, how the doping site controls charge storage, how ion transport and electronic properties determine kinetics, and how multielement strategies may provide further opportunities.

The identity of the dopant determines the type of structural and electronic perturbation introduced into the host MXene. Heteroatoms such as N can modify surface chemistry and create additional redox-active environments, whereas transition–metal dopants can directly alter the metal-centered electronic states [71]. This difference is evident for V_4_C_3_T_x_ and Nb_2_C. N incorporation into V_4_C_3_T_x_ through annealing in NH_3_ at 350 °C produced 210 F/g at 10 mV/s, representing a 17.3% improvement over pristine V_4_C_3_T_x_, with only 3.7% capacitance loss after 10,000 cycles [45]. The optimum result was associated with an intermediate N content and lower diffusion resistance. The effect of concentration is even clearer when the thermal treatment is varied. As evidenced by the XPS survey plots of V_4_C_3_T_x_, NH_3_-V_4_C_3_T_x_-350 °C and NH_3_-V_4_C_3_T_x_-550 °C, after NH_3_ annealing, an additional N 1s peak appears, confirming N incorporation (Figure 7a). The progressive decrease in F 1s intensity with increasing temperature indicates F removal, consistent with surface modification during N doping. Excessive N incorporation or aggressive annealing can remove surface terminations and promote structural changes that reduce electrochemical activity. Thus, increasing the dopant content does not necessarily increase capacitance. The same principle has been observed more broadly in doped MXenes, where excessive doping can reduce the accessible surface chemistry and deteriorate charge-storage behavior [72,73,74]. Metal doping can produce a prominent change in electronic structure [38,75,76]. Ni-doped Nb_2_C exhibited a specific capacitance of 666.67 F/g, compared with 258.6 F/g for pristine Nb_2_C, at 5 mV/s in PVA–H_2_SO_4_. The increase was attributed to additional electroactive sites and a higher density of states near the Fermi level. However, the electrode retained about 81% capacitance after 10,000 cycles, showing that the large increase in capacitance did not translate directly into superior long-term stability. These results suggest that an optimum dopant concentration should balance three factors: electronic conductivity, electrochemical activity, and structural stability. Too little doping may produce only a small electronic perturbation, whereas excessive doping may block active sites, disturb the MXene lattice, or introduce secondary phases.

**Figure 8:**
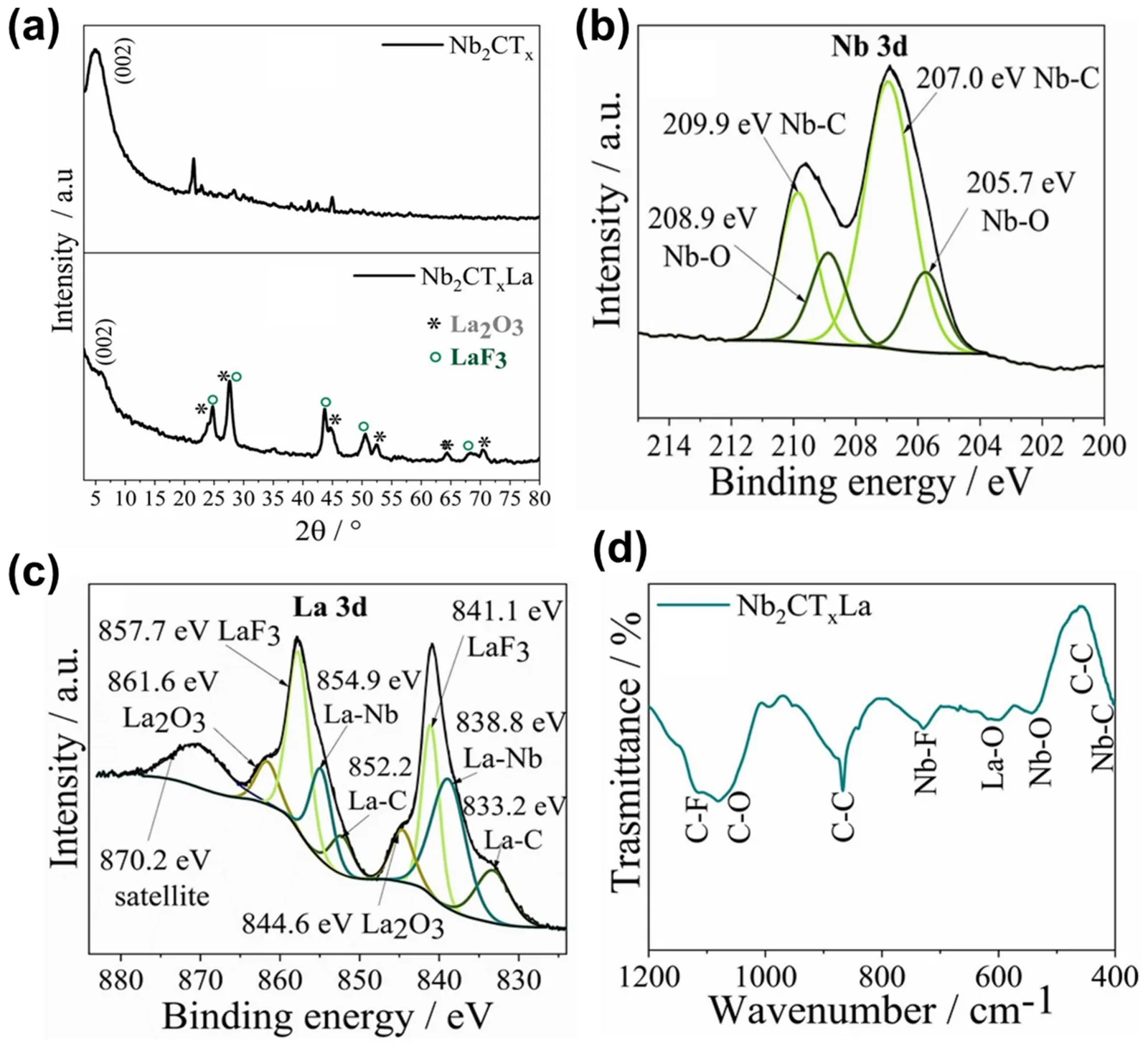
(**a**) XRD patterns of Nb_2_CT_x_ and Nb_2_CT_x_La, confirming the formation of La_2_O_3_ and LaF_3_ phases, (**b**) Nb 3d XPS spectra of Nb_2_CT_x_, (**c**) La 3d XPS spectra of Nb_2_CT_x_ La, and (**d**) FTIR spectrum of Nb_2_CT_x_La. Reprinted from ref. [70].

The location of the dopant is equally important. Doping in MXene can occur at M, X, or T sites, and each site produces a different type of chemical disruption. M-site substitution directly changes the transition–metal framework and therefore has a strong influence on d-orbital states, charge distribution, and redox activity. X-site doping changes the local M–X bonding environment, while T-site or surface modification mainly affects the electrode–electrolyte interface [77,78]. For non-Ti MXenes, experimental evidence is strongest for surface and heteroatom modification, whereas controlled M-site doping remains relatively limited. There are limited experimental verifications of M-site doping and a scarcity of X- and T-site studies as important gaps. This distinction is important because the same element may produce different electrochemical behavior depending on its location. The Ni-Nb_2_C example illustrates the role of metal-centered modification. Ni incorporation changed the total density of states and produced additional states near the Fermi level, which favoured electronic transport and charge storage [38]. La incorporation into Nb_2_CT_x_ and Nb_4_C_3_T_x_ MXene provides another useful example. La formed La–Nb and La–C bonds together with La_2_O_3_ and LaF_3_ phases [70]. The Nb 3d XPS spectrum confirms Nb–C and Nb–O bonds, whereas La 3d reveals La–Nb, La–C, La_2_O_3_, and LaF_3_ phase species (Figure 8a–c). FTIR further confirms Nb–C, Nb–O, Nb–F, La–O, C–C, C–O, and C–F bonds, supporting successful La incorporation and surface modification (Figure 8d). These changes increased interlayer spacing and generated additional redox-active sites. However, because secondary La-containing phases were also formed, the improvement cannot be assigned entirely to lattice La doping.

Doping improves supercapacitor performance only when the modified structure remains accessible to electrolyte ions. Increasing the interlayer distance can reduce the diffusion barrier and allow ions to reach otherwise inaccessible surfaces. At the same time, improved electronic conductivity reduces charge-transfer resistance and allows faster utilization of active sites. The N-Nb_2_CT_x_ system clearly demonstrates this relationship [67]. N doping increased the capacitive contribution from 31.1% to 64.64% and reduced the charge-transfer resistance from 1.43 to 1.20 Ω. Quantum capacitance provides another useful way to understand these changes. In MXenes, the total capacitance is influenced not only by ion adsorption at the interface but also by the electronic ability of the electrode to accommodate charge. Doping can alter the density of states near the Fermi level and therefore modify the quantum capacitance [79]. The broader doped-MXene literature has shown that changes in adsorption energy, electronic density, and surface active centers can increase quantum-capacitance contributions. For non-Ti MXenes, however, direct experimental separation of quantum capacitance from double-layer and pseudocapacitive contributions remains rare. This should therefore be treated as an important research gap rather than as an established mechanism for every doped non-Ti MXene.

Single-element doping changes one part of the MXene structure, whereas multielement or co-doping can introduce complementary effects [80,81,82,83]. One dopant may improve conductivity, while another can increase surface activity or modify interlayer spacing. Such a strategy is attractive for non-Ti MXenes because their broad compositional space allows multiple transition metals and heteroatoms to be combined. However, direct experimental evidence for co-doped non-Ti MXenes specifically in supercapacitors remains limited. The broader MXene literature demonstrates that N/S, N/P, and N/O combinations can provide synergistic changes in surface chemistry, active-site density, and charge storage [41,84,85,86,87,88]. These results provide a useful mechanistic basis for extending co-doping toward V-, Nb-, and Mo-based MXenes, but they should not be presented as direct evidence for non-Ti systems. A promising direction is therefore to combine two complementary dopants with controlled concentrations. For example, a transition–metal dopant could regulate the d-electronic structure while N, S, or P modifies the X-site or surface environment. Such designs could simultaneously increase electronic conductivity, create redox-active sites, expand ion–accessible regions, and stabilize the layered structure. The key challenge is to avoid excessive compositional complexity, which can make it difficult to identify which element is responsible for the observed improvement.

A direct comparison of doped non-Ti MXenes is difficult because reported capacitance values are obtained under different conditions. The specific capacitance of 210 F/g for N-doped V_4_C_3_T_x_ was obtained at 10 mV/s in 1 M H_2_SO_4_, whereas N-Nb_2_CT_x_ reached a specific capacitance of 640 F/g at 5 mV/s in an alkaline solution of 1 M KOH [45,67]. These differences in electrolyte, scan rate, voltage window, electrode mass loading, and cell configuration make simple ranking unreliable. In this respect, device-level metrics provide a more meaningful comparison. Cyclic stability also requires careful interpretation. La-incorporated Nb_4_C_3_T_x_ showed a remarkable 291% increase in capacitance after 90,000 cycles, but its coulombic efficiency fell to approximately 30% [70]. This suggests that the apparent capacitance increase may involve parallel unwanted reactions, such as water electrolysis, hydrogen/oxygen evolution, and electrode or electrolyte degradation, rather than only reversible charge storage. This example highlights why capacitance retention alone is insufficient for determining long-term stability. Coulombic efficiency, leakage current, self-discharge, impedance evolution, and post-cycling structural analysis should also be considered.

The structure-performance relationship of doped non-Ti MXenes in supercapacitors shows that the electrochemical benefit of doping is a multivariable phenomenon. Dopant identity controls the type of electronic and chemical perturbation, dopant concentration determines its magnitude, and dopant location governs whether the dominant effect occurs in the lattice, at the surface, or at the electrode–electrolyte interface. These changes subsequently influence ion diffusion, charge-transfer kinetics, redox activity, and electronic charge accommodation. For doped non-Ti MXenes, the most important unresolved issue is therefore not finding another dopant but establishing a predictive relationship between dopant location and electrochemical function. For meaningful comparison of doped MXene supercapacitors, high capacitance and apparent cyclic stability should not be considered sufficient evidence of improved charge storage. Reported performance should be supported by high Coulombic efficiency, reproducible charge–discharge behavior, and should be evidenced that unwanted Faradaic reactions, electrolyte decomposition, and electrode degradation do not dominate the measured charge-storage efficiency. Doped MXene-based electrodes showing poor Coulombic efficiency should therefore be treated as exhibiting significant unwanted electrochemical activity rather than actual enhancement of reversible charge storage mechanism. In addition, electrolyte chemistry should be treated as an essential parameter when evaluating doped non-Ti MXenes for supercapacitor applications. Changes in surface chemistry or dopant-induced redox activity may alter the electrode potential range, but the working device voltage must also satisfy the electrochemical stability of the electrolyte and electrode. Accordingly, electrochemical performance should be evaluated with the working voltage window, Coulombic efficiency, and long-term structural stability rather than from capacitance value only. The research should move toward controlled studies that combine spectroscopy, microscopy, electrochemical kinetics, DFT, and quantum-capacitance calculations. Such an approach would allow the actual contribution of doping to be separated from effects caused by secondary phases, surface functionalization, composites, and electrode architecture. This distinction will be essential for developing reliable design rules for next-generation doped non-Ti MXene supercapacitors.

## 7. Summary and Conclusions

Non-Ti MXenes provide a broad chemical platform for developing supercapacitor electrodes beyond the extensively studied Ti-based systems. This review examined Mo-, V-, and Nb- based MXenes and evaluated how their properties can be further tuned through M-, X-, and T-site modification, heteroatom incorporation, metal doping, substitution, and surface engineering. The discussion shows that the value of doping lies not simply in introducing a foreign element, but in using composition as a tool to control the electrochemical behavior of the host structure.

Several important conclusions emerge from the reviewed literature. First, doped non-Ti MXenes are still an emerging area for supercapacitors, despite the rapid growth of research on non-Ti MXenes in other energy-storage and conversion applications. Experimental studies remain concentrated mainly on V-, Nb-, and Mo-based systems, whereas many other non-Ti MXenes are still supported largely by theoretical predictions. Similarly, the lack of experimentally demonstrated S-, P-, and O-doped non-Ti MXenes for supercapacitors highlights an important gap that permits systematic investigation. Second, doping can provide a useful means of overcoming limitations associated with pristine MXenes by creating new chemical environments and modifying the accessibility of electroactive regions. However, the reported improvements should be interpreted in the context of the host MXene, electrolyte, electrode architecture, and synthesis conditions rather than through capacitance values alone. A central outcome of this review is the need to distinguish doping, substitution, surface functionalization, and composite formation. These terms are frequently used interchangeably, although they describe different structural situations and can lead to different electrochemical mechanisms. This distinction is particularly important for non-Ti MXenes because some reported performance enhancements originate from secondary phases or carbon-based components rather than from the dopant itself. The review also highlights that the current literature is not yet sufficient to establish universal rules for selecting a dopant or predicting its optimum concentration. Experimental determination of dopant location and distribution remains limited, especially for M-site doping, while systematic investigations of X- and T-site modification are even less common. Consequently, the field should move beyond empirical material screening toward controlled experiments in which composition, doping level, spatial distribution, and surface termination are deliberately varied. Another important conclusion concerns performance assessment. Electrode-level specific capacitance is useful for understanding intrinsic electrochemical behavior, but it does not by itself establish practical superiority. Energy density, power density, active-material loading, voltage window, coulombic efficiency, self-discharge, and long-term stability should be considered together. The unusual capacitance evolution reported for La-incorporated Nb_4_C_3_T_x_ further demonstrates why stability data require careful interpretation when parasitic reactions may contribute to the measured response.

Overall, doped non-Ti MXenes should be viewed as a design platform rather than a single class of high-capacitance materials. Their future development will depend on linking atomic composition with measurable electrochemical function and separating genuine doping effects from contributions arising from structure, surface chemistry, and electrode architecture. Combining controlled synthesis, advanced characterization, theoretical calculations, and standardized device testing can establish reliable design rules. Such progress would provide a stronger foundation for translating doped non-Ti MXenes from promising laboratory materials into practical high-power and high-performance supercapacitor electrodes. It is very important to note that the experimental evidence for doped non-Ti MXenes remains relatively limited, as many reported compositions and doping configurations have so far been explored mainly through theoretical studies. Consequently, N-modified VCT_x_ and NbCT_x_ appear more frequently in the present discussion because comparatively more experimental evidence is available for these systems. Their reported improvements should therefore be considered representative examples rather than trends for all doped non-Ti MXenes. Besides, experimental studies covering a wide range of compositions, dopants and well-defined doping configurations are needed to establish more general structure-property relationships for supercapacitor applications.

## 8. Challenges and Future Perspectives

Doped non-Ti MXenes have opened a broader compositional space for supercapacitor electrode design, but the field is still at an early stage. The main challenge is no longer to demonstrate that doping can change electrochemical behavior. Instead, the priority is to develop reproducible, scalable, and mechanistically controlled materials. The recent literature on non-Ti MXenes also shows that many compositions remain theoretically predicted, while experimental synthesis is concentrated mainly on V-, Mo-, and Nb-based systems.

**Controlled synthesis and dopant distribution:** Reliable control of dopant concentration, bonding state, and spatial distribution remains a major limitation. In particular, it is difficult to confirm whether a dopant occupies the M, X, or T position or forms a separate phase. Most theoretical studies focus on M-site substitution, whereas experimental verification of site-specific doping remains limited. Future work should combine controlled precursor chemistry to establish the actual dopant location. In situ characterization during synthesis would be particularly useful for following dopant incorporation and avoiding the common problem of assigning surface adsorption or secondary phases to lattice doping.

**Stability, oxidation, restacking, and scalable processing:** The practical use of non-Ti MXenes is also limited by oxidation, structural degradation, and restacking. Many non-Ti compositions have not yet been produced in large quantities, and their long-term stability in air and electrolyte environments is not well established. The broader non-Ti MXene literature identifies the development of air-stable materials and bulk synthesis as important requirements for practical applications. Doping may improve stability in some cases, but it can also introduce lattice strain or chemically unstable phases. The oxidation of the transition-metal centers can progressively alter the MXene surface and structure, which may trigger the loss of electrochemical activity and increased self-discharging. Additionally, hydrophobic functionalization and covalent ligand modification are possible approaches to restrict the possible contact with water and oxygen as well as improve environmental stability. Future studies should therefore evaluate storage stability, oxidation kinetics, and electrochemical degradation under realistic conditions rather than only measuring short-term cycling. Scalable preparation should also move toward safer etching, continuous processing, and high-yield delamination while maintaining the intended composition.

**Establishing genuine site-specific doping:** A major scientific gap is the lack of experimentally verified M-, X-, and T-site-specific doping. Theoretical calculations predict many attractive structures, but only a small fraction have been experimentally realized. This gap should be addressed by developing synthesis routes that selectively target individual sites. Such control would allow researchers to separate lattice effects from surface effects and determine whether a particular dopant improves conductivity, ion adsorption, redox activity, or structural stability. This information is essential for developing transferable design rules rather than relying on trial-and-error synthesis.

**Standardized electrochemical evaluation:** The current literature is difficult to compare because electrode loading, electrolyte, voltage window, scan rate, current density, and cell configuration vary substantially. Therefore, future reports should provide both intrinsic electrode properties and full-device performance. Mass loading should be clearly stated, and energy density should be calculated using the total mass of active materials where appropriate. Coulombic efficiency, self-discharge, leakage current, impedance evolution, and post-cycling structural changes should accompany cycling data. This is particularly important when capacitance increases during cycling, because such behavior may arise from irreversible reactions rather than genuine reversible charge storage. The reported studies suggest that doping can improve the electrochemical response of non-Ti MXenes, but the available studies do not yet support a universal trend. The observed enhancement depends on the dopant, its concentration and location, the host MXene, electrolyte, and electrode structure.

**From empirical screening to a technology roadmap:** The next stage should combine DFT, machine learning, automated synthesis, and experimental validation. DFT can first screen stable host–dopant combinations, preferred sites, formation energies, electronic structures, and ion–adsorption behavior. Experimental measurements can then validate the most promising candidates. A useful database should connect composition and synthesis parameters with dopant concentration, site occupancy, surface termination, conductivity, ion–transportation properties, capacitance, energy density, and cycling behavior. Machine-learning models could subsequently identify composition–structure combinations that are difficult to discover through conventional screening. Table 2 highlights the challenges, current limitations, and future research directions for doped non-Ti MXenes in supercapacitor applications, highlighting controlled synthesis, site-specific doping, structural stability, standardized electrochemical evaluation, and the transition toward data-driven and scalable material development.

A practical development pathway can therefore be divided into four stages: (i) computational screening and synthesis optimization, (ii) site-controlled material preparation and structural verification, (iii) standardized electrochemical and full-device testing, and (iv) scalable electrode fabrication and long-term validation. Flexible printing, coating, and filtration methods already demonstrated for non-Ti MXenes provide useful routes toward wearable and lightweight devices. The future of doped non-Ti MXene supercapacitors will depend on moving from composition–driven experimentation to mechanism-guided materials design. The most promising systems should not simply deliver high capacitance; they should combine controlled composition, oxidation resistance, scalable synthesis, rapid kinetics, high energy and power density, and reliable long-term operation. Establishing these links will determine whether doped non-Ti MXenes can progress from laboratory-scale demonstrations to practical high-performance supercapacitor electrodes.

## Figures and Tables

**Table 1:** Electrochemical energy storage performances of some non-Ti MXene–based electrodes for supercapacitor application.

Doped Non-Ti MXene–Based Systems	Electrolyte	Capacitance	Energy Density	Stability	Ref.
N-doped V_4_C_3_T_x_ (Three-electrode)	1 M H_2_SO_4_	210 F/g at 10 mV/s	–	96.3% after 10,000 cycles	[45]
N-doped Nb_2_CT_x_/rGO//N-doped Nb_2_CT_x_/rGO (Symmetric supercapacitor)	1 M TEABF_4_/ACN	152 F/g at 0.25 A/g	33 W h/kg	100% after 100,000 cycles	[66]
N-doped Nb_2_CT_x_//AC (Asymmetric supercapacitor)	1 M KOH	75 F/g at 1 A/g Deposited electrode mass: 1 mg	45 W h/kg	90% after 5000 cycles	[67]
N-doped Nb_2_CT_x_//AC (Asymmetric supercapacitor)	1 M aqueous (NH_4_)_2_SO_4_	66 F/g at 1 A/g Deposited electrode mass: 1.5–1.6 mg/cm^2^	23.36 W h/kg	92.31% after 12,000 cycles	[68]
N-doped Mo_2_C/C nanobelts	1 M LiClO_4_ in propylene carbonate, ethylene carbonate and dimethyl carbonate solution (a volume ratio of PC:EC:DMC of 1:1:1) with 5% fluoroethylene carbonate	1139 C/g at 1 mV/s	–	Over 90%	[69]
Ni-doped Nb_2_C MXene (Three-electrode)	1 M gel PVA–H_2_SO_4_	666.67 F/g at 5 mV/s	–	81% after 10,000 cycles	[38]
La-incorporated Nb_2_CT_x_ (2-electrode cell)		200.5 mF/cm^2^ at 5 mV/s	0.47 mW h/cm^2^	144.35% after 6000 cycles	[70]
La-incorporated Nb_4_C_3_T_x_ (2-electrode cell)		–	0.39 mWh/cm^2^	131% after 3000 cycles	[70]

**Table 2:** Challenges and future perspectives for doped non-Ti MXenes in supercapacitors.

Major Challenge	Current Limitations	Future Research Directions	Expected Outcome
Controlled synthesis and dopant distribution	Difficult to precisely control dopant concentration, bonding state, spatial distribution, and whether the dopant occupies M, X, or T sites or forms secondary phases.	Develop controlled precursor chemistry, site-selective synthesis, and in-situ characterizations of dopant incorporation.	Reproducible dopant concentration and location with clear structure–property relationships.
Stability, oxidation, restacking, and scalable processing	Non-Ti MXenes can undergo oxidation, structural degradation, and nanosheet restacking. Large-scale synthesis with consistent composition remains challenging.	Develop oxidation–resistant structures, protective coatings, interlayer engineering, safer etching, continuous processing, and high-yield delamination.	Improved long-term stability, reproducibility, and scalable production.
Establishing exact site-specific doping	Experimental evidence for M-, X-, and T-site-specific doping is limited, while many predicted structures remain experimentally unrealized.	Develop synthesis routes targeting specific sites and combine spectroscopy, microscopy, and DFT to verify dopant occupancy and bonding.	Site-specific design rules linking dopant location with conductivity, ion transport, redox activity, and stability.
Standardized electrochemical evaluation	Differences in mass loading, electrolyte, voltage window, scan/current rate, and cell configuration make direct comparison difficult.	Establish standardized testing protocols and report electrode– and full–device performance, including coulombic efficiency, self-discharge, leakage current, impedance, and post-cycling structure.	Reliable comparison of materials and more realistic assessment of practical performance.
From empirical screening to a technology roadmap	Trial–and–error material discovery is slow, and integrated databases connecting composition, structure, synthesis, and electrochemical performance are lacking.	Integrate DFT, machine learning, automated synthesis, and experimental validation to identify promising host–dopant combinations.	Faster discovery and rational design of high-energy, high-power, durable doped non–Ti MXene electrodes.

## Data Availability

No new data were created or analyzed in this study. Data sharing is not applicable to this article.
